# Computer Vision-Assisted Spatial Analysis of Mitoses and Vasculature in Lung Cancer

**DOI:** 10.3390/jcm14217526

**Published:** 2025-10-23

**Authors:** Anna Timakova, Alexey Fayzullin, Vladislav Ananev, Egor Zemnuhov, Vadim Alfimov, Alexey Baranov, Yulia Smirnova, Vitaly Shatalov, Natalia Konukhova, Evgeny Karpulevich, Peter Timashev, Vladimir Makarov

**Affiliations:** 1 Institute for Regenerative Medicine, Sechenov First Moscow State Medical University (Sechenov University), 8-2 Trubetskaya St., 119991 Moscow, Russia; fayzullin_a_l@staff.sechenov.ru (A.F.); timashev_p_s_@staff.sechenov.ru (P.T.); 2 Medical Informatics Laboratory, Yaroslav-the-Wise Novgorod State University, 41 B. St. Petersburgskaya, 173003 Veliky Novgorod, Russia; survial53@gmail.com (V.A.); zemnuhov2405@gmail.com (E.Z.); vadim.alfimov@novsu.ru (V.A.); baranovbik@mail.ru (A.B.); julka160502@gmail.com (Y.S.); vladimir.makarov@novsu.ru (V.M.); 3 Moscow State Budgetary Healthcare Institution “Moscow City Hospital named after S.S. Yudin, Moscow Healthcare Department”, Zagorodnoye Sh., 18A-7, 117152 Moscow, Russia; v.g.shatalov@yandex.ru; 4 Novgorod Regional Oncology Dispensary, 27 Lomonosov St., 173016 Veliky Novgorod, Russia; natalia.konjuhova@gmail.com; 5 Ivannikov Institute for System Programming of the Russian Academy of Science, Research Center for Trusted Artificial Intelligence, Alexander Solzhenitsyn St., 25, 109004 Moscow, Russia; karpulevich@ispras.ru

**Keywords:** lung cancer, deep learning, computational pathology, digital pathology, artificial intelligence, mitosis

## Abstract

**Background/Objectives:** Lung cancer is characterized by a significant microstructural heterogenicity among different histological types. Artificial intelligence and digital pathology instruments can facilitate morphological analysis by introducing calculated metrics allowing for the distinguishment of different tissue patterns. **Methods:** We used computer vision models to calculate a number of morphometric features of tumor vascularization and proliferation. We used two frameworks to process whole-slide images: (1) LVI-PathNet framework for vascular detection, based on the SegFormer architecture; and (2) Mito-PathNet framework for mitotic figure detection, based on the RetinaNet detector and an ensemble classification model. The results were visualized in the segmented and gradient heatmaps. **Results:** SegFormer for vessel segmentation achieved the following quality metrics: IoU = 0.96, FBeta-score = 0.98, and AUC-ROC = 0.98. RetinaNet + CNN ensemble achieved the following quality metrics: specificity = 0.96 and sensitivity = 0.97. The analysis of the obtained parameters allowed us to identify trophic patterns of lung cancer according to the degree of aggressiveness, which can serve as potential targets for therapy, including proliferative-vascular, hypoxic, proliferative, vascular, and inactive. **Conclusions:** The analysis of the obtained parameters allowed us to identify distinct quantitative characteristics for each histological type of lung cancer. These patterns could potentially become markers for therapeutic choices, such as antiangiogenic and hypoxia-induced factor therapy.

## 1. Introduction

Lung cancer remains one of the most significant medical and social challenges in modern oncology, ranking among the leading causes of morbidity and mortality worldwide [1]. Lung adenocarcinoma is the most common type of non-small-cell lung cancer, characterized by marked heterogeneity at the genetic and morphological levels [2]. Despite advances in diagnostic and therapeutic methods, the prognosis of the disease remains poor, necessitating the search for new therapeutic strategies.

The modern diagnosis of lung adenocarcinoma relies on an integrated approach that includes histological, immunohistochemical, and molecular genetic analyses. However, even with current diagnostic algorithms, certain “gray areas” persist, particularly in assessing the tumor grade, invasive potential, and tumor heterogeneity. In addition to well-established prognostic factors such as TNM stage and mutation status, increasing attention is being given to minor prognostic indicators, including features of angiogenesis, proliferative activity, and stromal-tumor interactions [3,4,5,6].

High microvascular density is strongly associated with lung cancer aggressiveness, progression, and post-operative recurrence [7]. Microvascular density is the clinical standard for evaluating tumor angiogenesis, which negatively affects the outcome of antitumor therapy with epidermal growth factor receptor-tyrosine kinase inhibitors (EGFR-TKI) [8].

Quantitative metrics of the tumor microenvironment, including the number and density of vessels in kidney cancer, were calculated using machine learning [9]; lymphovascular invasion (LVI) of breast cancer and colorectal cancer was also detected [10].

Mitotic score is a histologic marker of malignancy grade and its progression [11]. Mitoses detection is challenging both in digital pathology and manual examination, so instruments with high robustness are needed. An artificial intelligence (AI) model for the detection of breast cancer [12] mitoses has been developed, as well as a model for the detection of typical and atypical mitoses in colorectal cancer [13].

The role of mitoses in lung cancer is less standardized, and the spatial relationships of mitotic figures with other components of the tumor microenvironment have not been considered.

In this study, we tested the hypothesis that the quantitative metrics of the vascular network and mitotic figures can be indicative of histological types of lung cancer. To test this hypothesis, we applied two computer vision networks and interpreted the results with thorough morphological examination.

## 2. Materials and Methods

**Figure 1:**
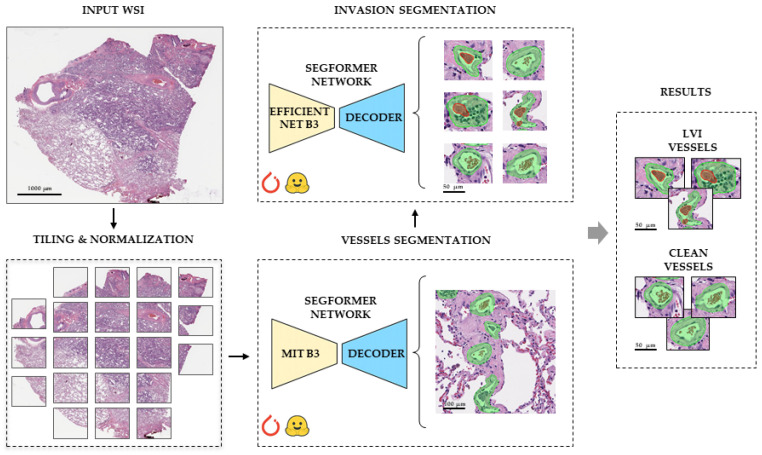
Overall scheme of the modified LVI-PathNet framework for automated detection of LVI on WSIs. The pipeline begins with an input WSI, which undergoes tiling and normalization. This is followed by a two-stage semantic segmentation process. First, vessel segmentation (utilizing a SegFormer network with a Mit B3 backbone and a decoder) identifies vessel structures (green masks). Subsequently, invasion segmentation (utilizing a SegFormer network with an EfficientNet B3 backbone and a decoder) processes these identified vessels to detect tumor cells within them (red regions inside green masks). The final results clearly distinguish LVI vessels from clean vessels. Scale bars—1000, 100, and 50 µm.

**Figure 2:**
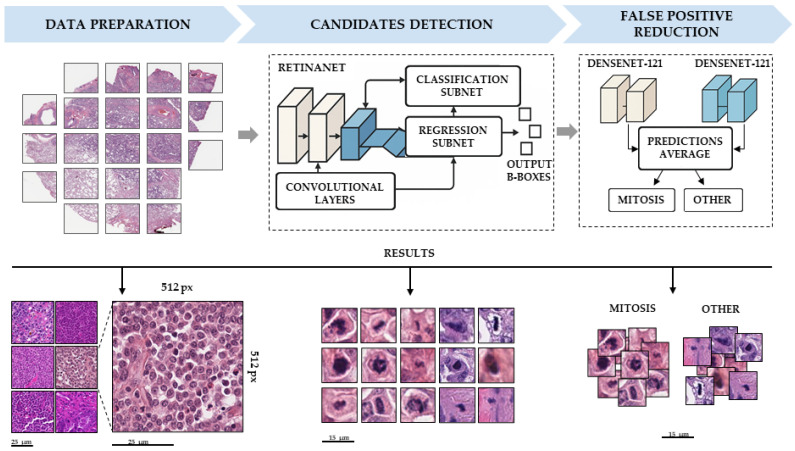
Mito-PathNet framework workflow: a two-stage deep learning pipeline for mitotic figure localization. The pipeline includes data preparation, where WSIs are divided into 512 × 512 pixel tiles. The candidates detection stage utilizes the RetinaNet architecture to identify potential areas of interest and generate bounding boxes. Subsequently, the false positive reduction stage is implemented as an ensemble of two DenseNet-121 convolutional neural networks, which classifies objects as “mitosis” or “other.” The lower part of the diagram illustrates representative examples of inputs and outputs at each stage. Scale bars—15 and 25 µm.

To detect blood vessels, a modified version of the LVI-PathNet framework was used [14], which is a two-stage semantic segmentation system for automated detection of LVI on whole-slide images (WSIs) (Figure 1). The Mito-PathNet framework (Figure 2) is a two-step deep learning pipeline, including (1) preliminary identification of mitotic figures and (2) refined classification (false positive reduction mechanism).

### 2.1. Data Preparation

Comprehensive data preparation was performed to train the LVI-PathNet models. Publicly available datasets included the following:

1. DHMC (Dartmouth-Hitchcock Medical Center), a collection of 143 WSIs of lung adenocarcinoma from the Dartmouth-Hitchcock Pathology Center [15].
2. NLST (National Lung Screening Trial), a large-scale clinical trial involving 451 WSIs from lung cancer patients enrolled in the NLST screening study (total cohort: 1225 WSIs) [16].
3. TCGA-LUAD (The Cancer Genome Atlas Lung Adenocarcinoma), a collection containing 150 WSIs of lung adenocarcinoma, each paired with clinical, genomic, and radiologic metadata from the TCGA consortium [16].

Additionally, our own dataset, OWN_DB, was compiled, containing 56 WSIs sourced from regional cancer centers, including the Novgorod Regional Dispensary, City Clinical Hospital named after S.S. Yudin, and Sechenov University. In total, 199 WSIs of lung adenocarcinoma were prepared and annotated. Dataset composition statistics are presented in Table 1. The final training set captures the diversity of WSI scanning protocols, H&E staining variations, and the morphological heterogeneity of lung tumor tissue.

The MIDOG++ dataset served as the primary source for training and validating the Mito-PathNet framework. This collection comprises 503 histological images (∼7000 × 5000 pixels each) and seven tumor types (human breast cancer, neuroendocrine tumors, melanoma, canine lung cancer, lymphoma, mastocytoma, and soft tissue sarcoma).

### 2.2. Annotation

The annotation of vascular structures and invasion sites was performed manually by pathologists. In total, 17,882 vessels were annotated, of which 17,357 were classified as “clean” (no signs of invasion) and 525 as invasive. To train the segmentation model for cancer cell clusters inside vessels, 707 LVI sites were annotated across the 525 vessels with invasion.

Mitosis annotations were derived from independent assessments by two expert pathologists from the MIDOG++ dataset [17], with a third expert resolving discrepancies to establish consensus labels. Final training data included 11,917 mitosis-positive patches and 14,351 hard-negative patches (challenging false positives).

### 2.3. Training, Test, and Validation

#### 2.3.1. Vessel Segmentation

To increase the performance of LVI detection, the pipeline was developed for each deep learning model from the LVI-PathNet framework, including data preparation, training, and validation.

**Figure 3:**
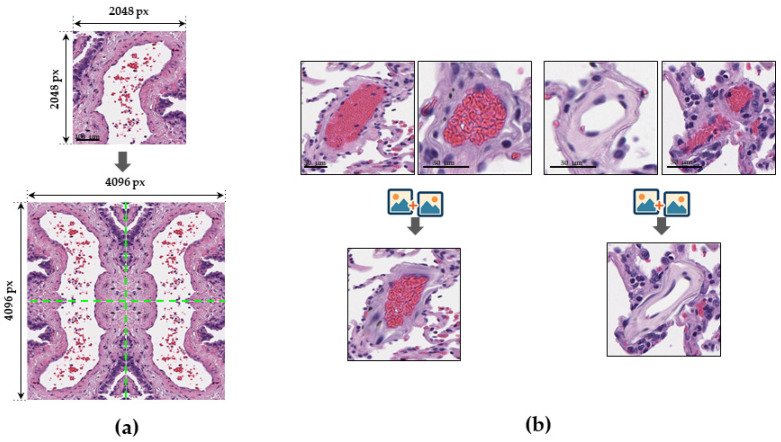
Examples of data augmentation strategies. (**a**) The ButterflyReflectionPattern method, demonstrating an image (**top**) mirrored horizontally and vertically to form a larger, symmetrical representation (**bottom**). Scale bar—100 µm. (**b**) Illustration of synthetic example generation: a non-invasive vessel segment (**top left**) is replaced with an invasive segment (**top right**) to create a new composite image (**bottom**). Scale bar—100 and 50 µm.

A SegFormer [18] model with a MiT-B3 encoder was used for vessel segmentation. Human lung adenocarcinoma WSIs were split into 70% training, 15% validation, and 15% testing for each of the five-fold cross-validation iterations. To expand data and address class imbalance, WSIs were divided into 2048 × 2048 px tiles, then downscaled to 512 × 512 px. Three tiling methods were employed: BBox tiling (vessel-centered bounding boxes), Mask tiling (fixed-size tiles for context) and Grid shift (shifted bounding boxes). In total, 265,341 images were obtained per fold, distributed as 185,739 for training, 39,801 for validation, and 39,801 for testing. Color channels were normalized using ImageNet’s mean/std. Spatial and color augmentations (rotations, flips, and brightness/contrast/saturation adjustments) were applied. A custom ButterflyReflectionPattern transformation [19] (Figure 3a) minimized tile boundary artifacts. The model was trained with a modified Combo Loss, combining Weighted Cross Entropy and Dice Loss for robust segmentation. Each model was trained on a five-fold split.

A SegFormer model with an EfficientNet B3 encoder was used for the invasion segmentation. The dataset was split into 80% training, 10% validation, and 10% testing across five-fold cross-validation iterations. To train for intravascular cancer cells, 707 LVI sites across 525 invasive vessels were annotated. A total of 50,000 synthetic tiles were generated by realistically replacing non-invasive vessel segments with invasive ones to overcome the lack of invasion tiles (Figure 3b). The BBox, Mask, and Grid shift methods were also used for WSI tiling. Data preparation also included normalizations and various augmentations (rotations, flips, brightness/contrast adjustments) to enhance robustness. Training utilized a semantic segmentation-optimized loss function, likely combining Dice Loss and Cross-Entropy Loss, to effectively detect complex invasion structures.

#### 2.3.2. Mitosis Detection

For data preparation, the MIDOG++ dataset (503 WSIs) [17] was systematically split into 392 (77.9%) WSIs for training and 111 (22.1%) WSIs for testing. All WSIs were divided into 512 × 512 pixel tiles at the highest magnification level.

Customized RetinaNet [20,21] was trained to detect mitotic figures. Training involved stratified five-fold cross-validation on the training set. Each epoch sampled 1000 training and 250 validation patches using a guided strategy: 50% randomly and 50% close to annotated ROI for slides with mitotic figures. Input patches underwent z-score normalization based on the mean and standard deviation of the tissue-containing areas of their respective training images. Standard Fastai v1 augmentations (random rotations, flipping, affine transformations, and color jitter) were applied. Models were trained for 120 epochs (batch size 20, discriminative learning rate [5 × 10^−5^, 5 × 10^−4^]) with standard RetinaNet loss (smooth L1 for regression, focal for classification). The model with the highest AP in the validation set was selected retrospectively.

**Figure 4:**
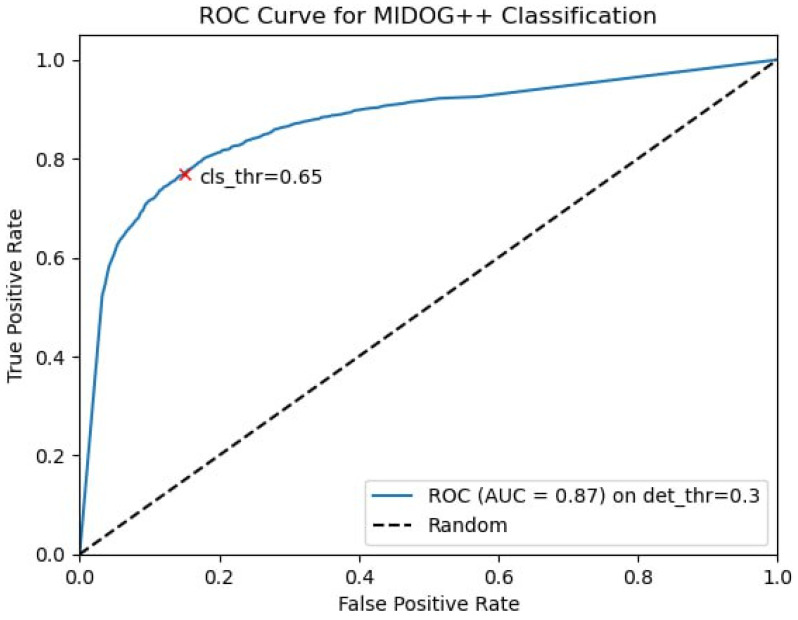
Classification step ROC curve. The AUC of 0.87 indicates strong classification performance, with optimal results achieved at a threshold of 0.65.

To improve Retina’s performance, we introduced an ensemble of two CNN DenseNet121 [21] as a second classification step to decrease the detector’s threshold to obtain more true positives (TPs) and false positives (FPs). The filtration of the FP was performed at the second step with a more precise classification. During model verification on field data (human lung adenocarcinoma), we discovered other types of FPs from coal dust (anthracosis), ink, muscle/fibroblast nuclei, and chromatin parts in necrosis. To overcome this, we enhanced the classification model training set with 918 anthracosis images, 1177 ink images, 587 muscle/fibroblast nuclei, and 278 chromatin part images on 24 WSIs. The dataset was split into 16 training and 8 testing WSIs, so the proportion of train/test went from 2:1 to 3:1 for each FP class. Then we added these images into the source dataset and retrained the classification model, reaching the optimal threshold for the detection and classification steps: 0.3 and 0.65, respectively (Figure 4).

For inference and evaluation on the 111 test WSIs, both the RetinaNet and the RetinaNet + Ensemble pipeline were tested. A sliding-window approach (10% overlap) and non-maximum suppression were used to process detections. Mitotic figure candidates were then evaluated against ground truth annotations from the MIDOG++ dataset and the mean F1 score was computed across all test WSIs.

Different WSIs were used to train and test the model. That means that the same slide was not included in both the training and test samples at the same time. Since each WSI corresponded to a separate patient, the slide separation automatically ensured that there was no overlap of patients between the samples.

### 2.4. Morphology, Parameters, and Metrics

**Figure A1:**
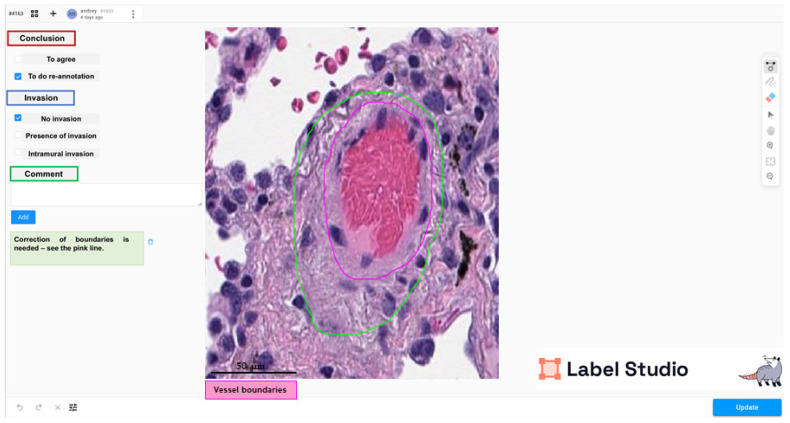
Validation of the primary annotations was performed in Label Studio. The experts evaluated each annotation separately and made a conclusion on it (red rectangle): “to agree” or “to do re-annotation”. The presence or absence of invasion was evaluated as a separate feature (blue rectangle): “no invasion”, “presence of invasion”, or “intramural invasion”. At the end of the validation, a comment was given (green rectangle) and, if necessary, the expert used the brush tool to draw new annotation boundaries (pink line) to replace the previous annotation (green line).

AI-generated masks were manually and thoroughly reviewed by a pathologist with three years of experience. The patterns of vascularization (vessel distribution in the tumor and in the area around the tumor, and the predominant caliber of vessels) and proliferation (mitosis distribution in the tumor area, hot spots, and formation of clusters by mitoses around the vessels), as well as the presence of necrosis were described for 207 cases of lung adenocarcinoma from the DHMC dataset, TCGA-LUAD dataset, and the OWN_DB dataset (15 per micropapillary subtype, 84 per solid subtype, 13 per papillary subtype, 21 per lepidic subtype, and 74 per acinar subtype). All initial masks for vessels, LVI, and reports were then independently verified by two senior pathologists. In cases of disagreement, a consensus adjudication process was used to establish the final ground truth (interobserver variability was calculated using the Dice metric and achieved 0.92 for vessels). This material was added to the Appendix A to show the expert validation (Figure A1). In approximately 20% of the cases, the MIDOG++ experts disagreed in their assignment of mitotic and non-mitotic figures [17]. For domain adaptation for human lung cancer (DHMC), we enriched annotations with anthracosis, chromatin, and ink patches. Then, the following regions of interest were annotated: the tumor zone and area surrounding the tumor (with 1:1 area on each WSI). We created two datasets for analysis: “native” with uncorrected AI-generated masks and “clean” with corrected AI-generated masks. After extracting the JSON, we calculated the primary morphological features (Table A1 Appendix A) and vascularization-proliferation metrics (Table A2 Appendix A).

The results were visualized in the segmented and gradient heatmaps showing the intensity of blood supply in the tumor and around the tumor, as well as the foci of tumor proliferation.

For scanner magnification-independent vessel diameter count, the Feret diameter approach was used. Vessels were divided into “large” and “small” according to the 300 µm^2^ threshold. This justification was based on the established biological definitions from prior literature [14]. We clarify that this threshold was chosen to meaningfully separate small capillaries from larger venules/arterioles for our biological inquiry. Minimum and maximum vessel diameters were detected by the rotating calipers method, described by Shamos [22].

All parameters and metrics were performed on Python 3.12. The statistical analysis of the experimental quantitative data was performed with a standard program package, GraphPad Prism version 10.00 for Windows (GraphPad Software, Inc., San Diego, CA, USA). Intergroup differences in quantitative data were analyzed by the two-way ANOVA method (column factor was 2—“clean” and “native”, and row factor was 5–5 histologic subtypes of lung adenocarcinoma). Distribution normality of data was determined by the Shapiro–Wilk test (α > 0.05). The results of the statistical analysis were presented as column graphs of the mean values and standard deviations (SDs) for quantitative data. *p*-values ≤ 0.05 were considered statistically significant. Differences between column means for each vascularization-proliferation metric were also reported (Table A3 Appendix A).

## 3. Results

Using SegFormer for segmentation of human lung cancer cell groups inside blood vessels achieved the quality metrics of IoU = 0.96, FBeta-score = 0.98, and AUC-ROC = 0.98. The model marked the vessels with smooth masks of green (vessels without signs of invasion) and green with red tumor cells inside (vessels with signs of invasion). The boundaries of the masks captured a small part of the connective tissue fibers of tunica adventitia. The model distinguished vessels of large, medium, and small caliber equally well. Masks of arterioles, venules, and capillaries resembled circles and ovals, while masks of vessels of larger calibers could merge due to the common tunica adventitia into one irregular shape. The model correctly identified tumor invasion both in tunica media and directly in the lumen of vessels of various diameters. There were cases of false positive detections when the connective tissue fibers of the alveoli were also marked with small green masks with ragged edges. In addition, single bronchial epithelial cells were classified as tumor cells.

RetinaNet + CNN ensemble achieved quality metrics of precision = 0.89, sensitivity = 0.83, and F1-score = 0.86 on the MIDOG++ test set and metrics of specificity = 0.49 and sensitivity = 0.92 on the DHMC test set (Table 2). The combined model highlighted the mitosis figures with square semi-transparent yellow masks. Both typical mitoses in all phases and atypical mitoses were equally well defined. There were also false positive detections of muscle/fibroblast nuclei and chromatin parts in necrosis. After tuning, the ensemble achieved quality metrics of specificity = 0.96 and sensitivity = 0.97 on the DHMC test set (Table 2).

**Figure 5:**
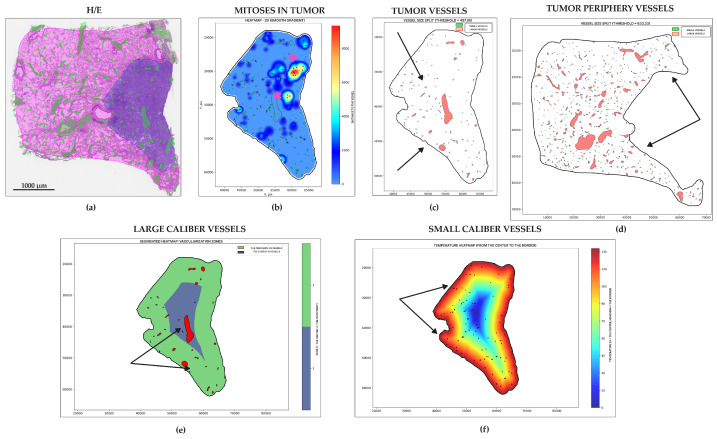
Micropapillary lung adenocarcinoma, WSIs N = 15. (**a**) Whole-slide image (WSI) with annotations for tumor (purple) and peritumoral tissue (pink), and vessel segmentation (green) and mitotic figures (yellow squares). (**b**) Mitotic heatmap reveals a diffuse and high proliferation rate throughout the tumor (hot spots indicated by star). (**c**,**d**) Vessel segmentation masks within the tumor (**c**) and peritumoral tissue (**d**). Large (red) and small (green) caliber vessels are predominantly located at the tumor periphery (arrows). (**e**) Segmented heatmap of large vessels confirms their scarcity in the tumor center and abundance at the periphery. (**f**) Gradient heatmap of small vessels shows a dense vascular network at the tumor periphery (arrows). Scale bar—1000 µm.

**Figure 6:**
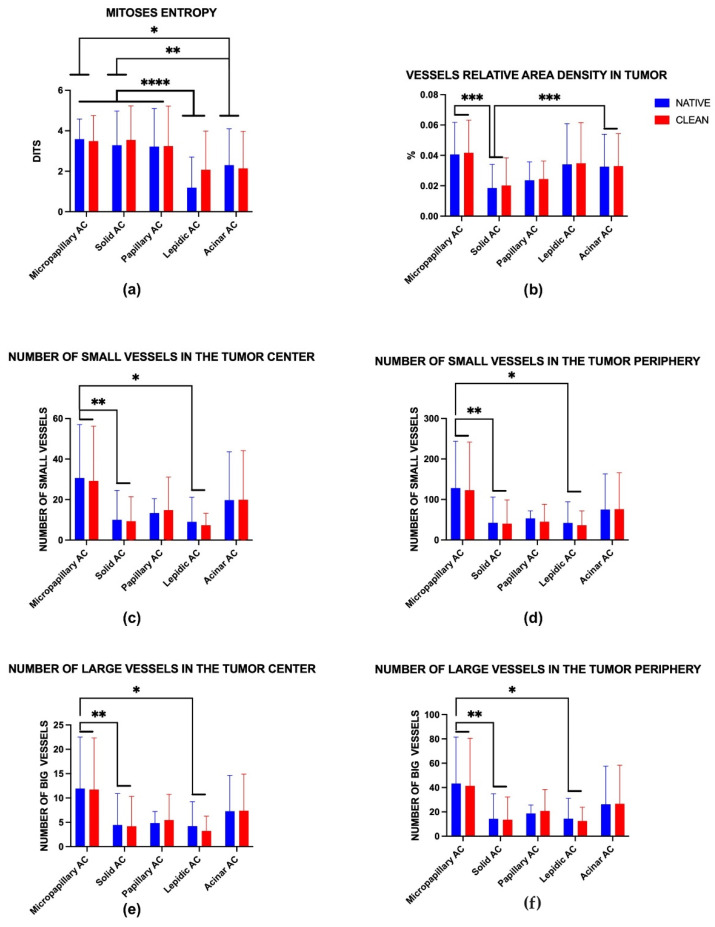
Statistical analysis of mitoses and vasculature in WSIs N = 207 of lung adenocarcinoma subtypes: micropapillary adenocarcinoma n = 15, solid adenocarcinoma n = 84, papillary adenocarcinoma n = 13, lepidic adenocarcinoma n = 21, and acinar adenocarcinoma n = 74. (**a**) Mitoses entropy, mean ± SD, dits. (**b**) Vessels relative area density in tumor, mean ± SD, %. (**c**) Number of small vessels in the tumor center, mean ± SD, units. (**d**) Number of small vessels in the tumor periphery, mean ± SD, units. (**e**) Number of big vessels in the tumor center, mean ± SD, units. (**f**) Number of big vessels in the tumor periphery, mean ± SD, units. Star (*)—*p*-value ≤ 0.05, stars (**)—*p*-value ≤ 0.01, stars (***)—*p*-value ≤ 0.001, stars (****)—*p*-value ≤ 0.0001.

Micropapillary adenocarcinoma (Figure 5a) showed the highest level of mitoses entropy (3.59 ± 0.99 dits) (Figure 5b and Figure 6a) and the highest number of vessels in the tumor centers (30.60 ± 26.44 units for small, 11.93 ± 10.59 units for large) (Figure 5e,f and Figure 6c,e) and peripheries (128.00 ± 115.10 for small, 43.40 ± 38.11 units for large) (Figure 5d and Figure 6d,f). It was also a leader in vessel relative area density in tumors (0.05 ± 0.02%) (Figure 5c and Figure 6b). Morphologically, it appeared as a diffuse chaotic proliferation in the whole tumor with a rich vascular net.

**Figure 7:**
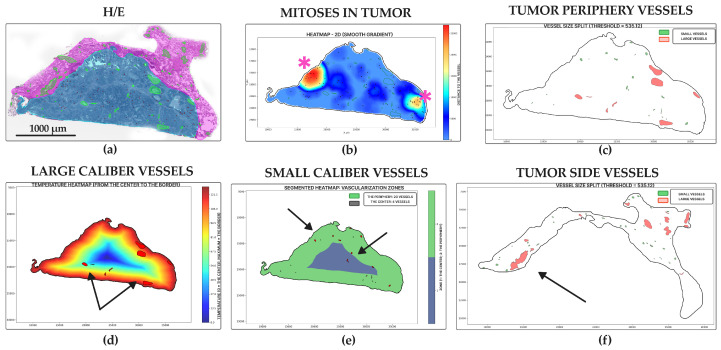
Solid lung adenocarcinoma, WSIs N = 84. (**a**) Annotated WSI showing tumor (purple), peritumoral tissue (pink), vessels (green), and mitotic figures (yellow squares). (**b**) Mitotic heatmap shows a high, diffuse proliferation pattern with pronounced hot spots at the tumor periphery (star). (**c**) Sparse distribution of both large (red) and small (green) caliber vessels within the tumor, primarily at the periphery. (**d**) Gradient heatmap of large vessels indicates their low density, restricted to the tumor periphery (arrows). (**e**) Segmented heatmap of small vessels shows a scant distribution both centrally and peripherally. (**f**) Peritumoral tissue contains mainly small-caliber vessels, with occasional large vessels (arrow). Scale bar—1000 µm.

Solid adenocarcinoma (Figure 7a) had a high level of mitoses entropy (3.28 ± 1.68 dits) (Figure 6a and Figure 7b), almost like micropapillary adenocarcinoma, and the lowest number of vessels in the tumor center (10.03 ± 14.48 units for small, 4.44 ± 6.45 units for large) (Figure 6c,e and Figure 7e,f) and periphery (42.59 ± 63.15 for small, 14.26 ± 20.64 units for large) (Figure 6d,f and Figure 7d). It had the lowest vessel relative area density in tumors (0.01 ± 0.01%) (Figure 6b and Figure 7c). Each WSI showed large areas of necrosis in the whole tumor and a lack of vascular net both in the tumor and its periphery.

**Figure 8:**
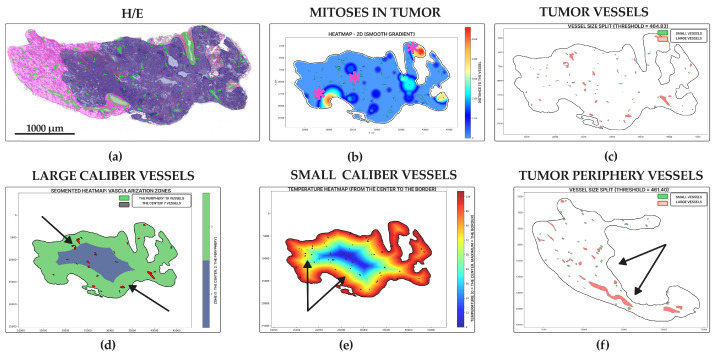
Acinar lung adenocarcinoma, WSIs N = 74. (**a**) Annotated WSI: tumor (purple), peritumoral tissue (pink) with overlays for vessels (green) and mitoses (yellow squares). (**b**) Mitotic heatmap displays a focal, moderate proliferation level, with hot spots often associated with vessels (star). (**c**) Moderate density of large (red) and small (green) caliber vessels, with a slight predominance at the tumor periphery. (**d**) Segmented heatmap shows a few large vessels distributed both centrally and peripherally (arrows). (**e**) Gradient heatmap reveals a moderate network of small vessels, primarily at the tumor periphery (arrows). (**f**) Peritumoral vasculature consists mostly of small vessels, with rare large ones (arrow). Scale bar—1000 µm.

Acinar adenocarcinoma (Figure 8a) was the second leader in vascularity; we identified a high number of vessels in the tumor center (19.71 ± 23.87 units for small, 7.27 ± 7.35 units for large) (Figure 6c,e and Figure 8e,f) and periphery (75.04 ± 88.18 for small, 26.28 ± 31.37 units for large) (Figure 6d,f and Figure 8d). Low levels of mitoses entropy (2.30 ± 1.80 dits) indicated the formation of clusters by mitoses (Figure 6a and Figure 8b). It showed the second-highest vessel relative area density in tumors (0.03 ± 0.02%) (Figure 6b and Figure 8c). Histologic specimens demonstrated tumor glands with mucin (sometimes), diffuse proliferation with several numbers of mitoses clusters, especially around vessels—“hot spots”.

**Figure 9:**
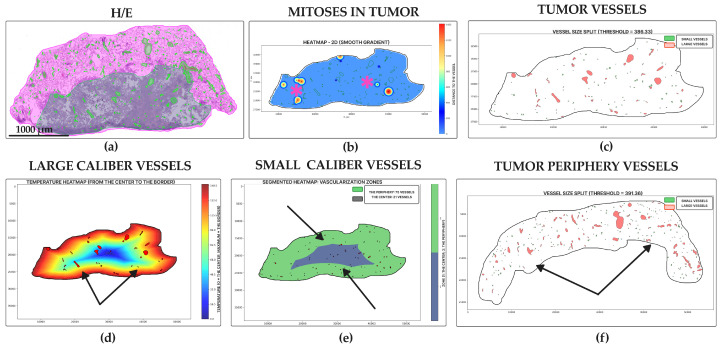
Papillary lung adenocarcinoma, WSIs N = 13. (**a**) Papillary lung adenocarcinoma annotated WSI with tumor (purple), peritumoral tissue (pink), segmented vessels (green), and detected mitoses (yellow squares). (**b**) Mitotic heatmap indicates a low-to-moderate, diffuse proliferation with isolated hot spots (star). (**c**) Moderate, uniform distribution of large (red) and small (green) caliber vessels within the tumor. (**d**) Gradient heatmap shows an even distribution of large vessels throughout the tumor (arrows). (**e**) Segmented heatmap confirms a homogeneous distribution of small vessels (arrows). (**f**) Peritumoral tissue contains a mix of both large and small caliber vessels (arrows). Scale bar—1000 µm.

Papillary adenocarcinoma (Figure 9a) showed one of the highest levels of mitoses entropy (3.22 ± 1.88 dits) (Figure 6a and Figure 9b) and a moderate number of vessels in the tumor center (13.30 ± 7.14 units for small, 4.84 ± 2.37 units for large) (Figure 6c,e and Figure 9e,f) and periphery (53.308 ± 18.781 units for small, 18.69 ± 7.02 units for large) (Figure 6d,f and Figure 9d). Vessel relative area density in tumors also took a joking position (0.02 ± 0.01%) (Figure 6b and Figure 9c). Tumors consisted of papillary structures with significant vascular cores and had a moderate diffuse proliferation with formation of singular hot spots.

**Figure 10:**
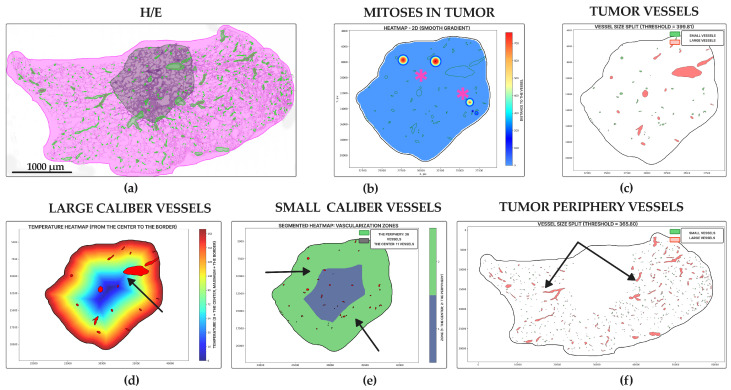
Lepidic lung adenocarcinoma, WSIs N = 21. (**a**) Annotated WSI of lepidic lung adenocarcinoma (purple) with peritumoral regions (pink), vessel masks (green). and mitotic figures (yellow squares). (**b**) Mitotic heatmap shows a low, focal proliferation pattern with isolated hot spots (star). (**c**) Sparse and uniform distribution of large (red) and small (green) caliber vessels within the tumor. (**d**) Gradient heatmap of large vessels shows their scarce and even distribution (arrow). (**e**) Segmented heatmap confirms a homogeneous, low-density distribution of small vessels (arrows). (**f**) Peritumoral tissue contains a mixture of large and small caliber vessels (arrows). Scale bar—1000 µm.

Lepidic adenocarcinoma (Figure 10a) showed the lowest level of mitoses entropy (1.19 ± 1.51 dits) (Figure 6a), which morphologically appeared as singular mitotic clusters (Figure 10b), and the lowest vascularization both in the tumor and at the tumor periphery (Figure 10c–f). It had a lack of vascular nets: the number of small vessels was 9.04 ± 2.08 units (tumor center) and 42.04 ± 52.54 units (tumor periphery) (Figure 6c,d), and the number of large vessels was 9.22 ± 5.01 units (tumor center) and 14.42 ± 16.74 units (tumor periphery) (Figure 6e,f). Additionally, the tumor area vessels relative density was similar to that of in acinar carcinoma—0.03 ± 0.02% (Figure 6b).

## 4. Discussion

**Figure 11:**
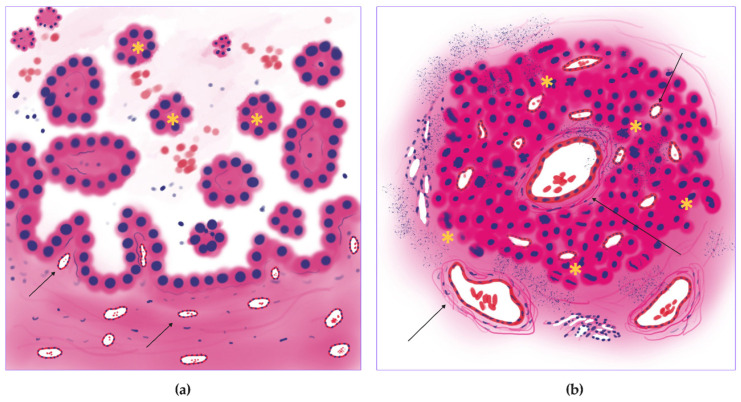
Proliferative-vascular trophic pattern of the micropapillary lung adenocarcinoma. (**a**) Schematic image of the micropapillary lung adenocarcinoma with small vessel vascular net (arrows) and micropapillae (stars). (**b**) Matching histologic subtype image of the proliferative-vascular pattern with diffuse mitotic activity (stars) and high vascular density, especially small vessel caliber (arrows).

**Figure 12:**
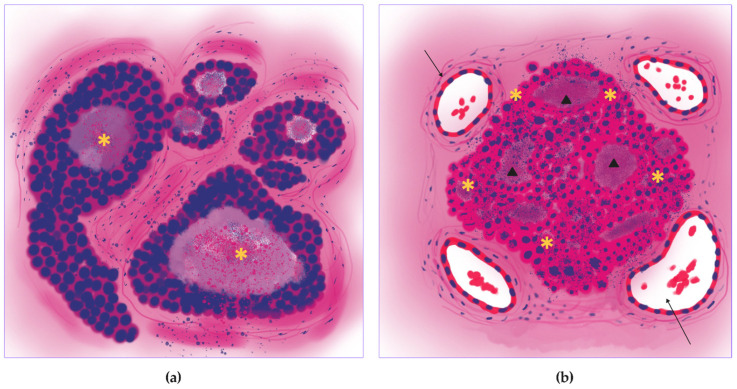
Hypoxic trophic pattern of the solid lung adenocarcinoma. (**a**) Overall scheme of the solid adenocarcinoma with necrosis in tumor nests (stars). (**b**) The image of the proposed hypoxic pattern with diffuse mitotic activity (stars), necrosis (arrowhead), and high vascular density on the tumor periphery (arrows).

**Figure 13:**
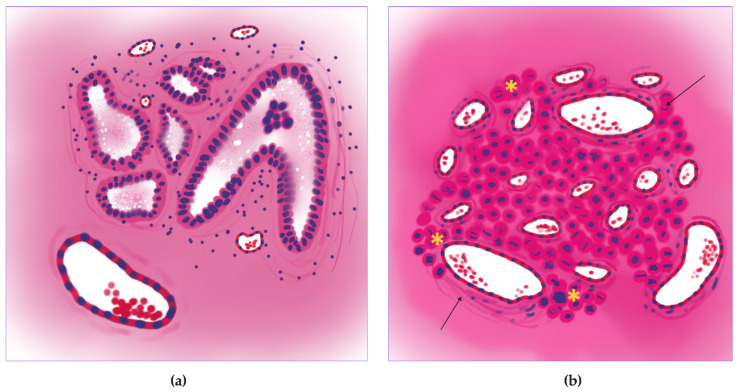
Proliferative trophic pattern of the acinar lung adenocarcinoma. (**a**) The acinar lung adenocarcinoma with irregularly shaped glands, containing mucin. (**b**) Illustration of the proliferative pattern with mitotic activity around intratumor vessels (stars), and blood supply in the tumor center and on the tumor periphery mainly with small caliber vessels (arrows).

**Figure 14:**
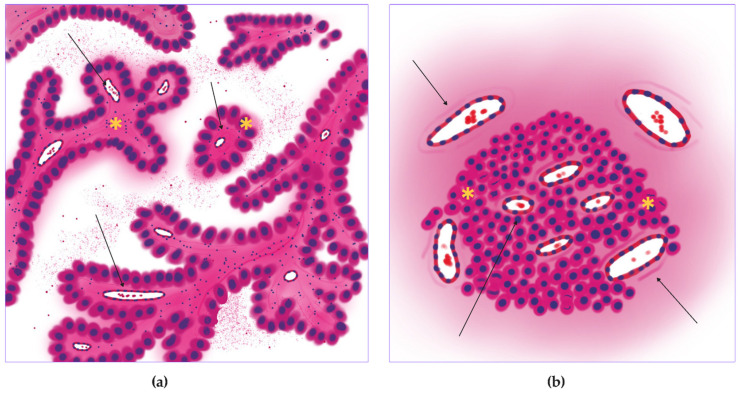
Vascular trophic pattern of the papillary lung adenocarcinoma. (**a**) Key features of the papillary lung adenocarcinoma: papillae (stars) showing true fibrovascular cores (arrows). (**b**) Schematic image of the vascular pattern with moderate diffuse mitotic activity (stars), and balanced center/periphery blood supply with both large and small vessels (arrows).

**Figure 15:**
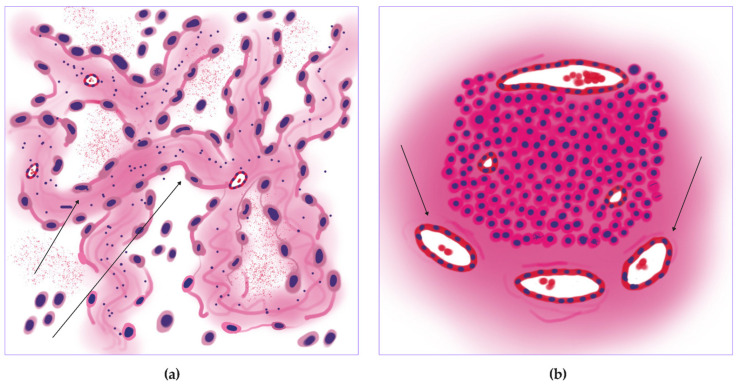
Inactive trophic pattern of the lepidic lung adenocarcinoma. (**a**) The lepidic lung adenocarcinoma shows tumor cells growing along the walls of the alveoli (arrows). (**b**) Overall scheme of the inactive pattern with low/absent mitotic activity and mainly peripheric vascularization, predominantly by small vessels (arrows).

In this study, we described distinct trophic patterns for each subtype of lung adenocarcinoma. (1) Micropapillary lung adenocarcinoma had high mitotic activity throughout tumor tissue and dual blood supply with central and peripheral vascularization (proliferative-vascular pattern) (Figure 11). (2) Solid lung adenocarcinoma had high mitotic activity, peripheral vascular dominance, and significant necrotic areas (hypoxic pattern) (Figure 12). (3) Acinar lung adenocarcinoma was characterized by combined central/peripheral vascularization and mitotic clustering around intratumoral vessels (proliferative pattern) (Figure 13). (4) Papillary lung adenocarcinoma had balanced vascular supply and moderate mitotic activity (vascular pattern) (Figure 14). (5) Lepidic lung adenocarcinoma had minimal/absent mitotic activity and predominantly peripheral vascularization (inactive pattern) (Figure 15). These trophic patterns reflect the functional features of the tumor metabolism (vascularization and hypoxia) and tumor growth intensity (proliferative activity, LVI, and metastasis).

The main finding of the article is that combined analysis of tumors’ trophic behaviors allows for a stronger quantitative comparison of histological subtypes of lung adenocarcinoma than a separate study of blood vessels [10,14] or mitoses [12,13]. It also presents an insight into hypoxia-inducible factor (HIF) mechanisms and the availability of drugs through the bloodstream. It is known that use of HIF therapy and other targeted methods depends on the histological subtype of lung adenocarcinoma and its molecular features [23]. Although hypoxia is typical for many solid tumors, the effectiveness of HIF-directed approaches may vary depending on the genetic profile of the tumor.

Some inhibitors are universal and affect every subtype of lung adenocarcinoma with hypoxia presence: PX-478 inhibits HIF-1α translation by blocking its interaction with the ribosome and also increases p53 and reduces c-Myc translation; and EZN-2968 targets HIF-1α mRNA, leading to decreased HIF-1 protein levels [18,19]. HIF-therapy strategies depend on genetic mutations (epidermal growth factor receptor—EGFR) and biomarker expression programmed death-ligand 1 (PD-L1) [24]; high levels of EGFR mutations with low PD-L1 expression cause resistance both to immunotherapy and chemotherapy.

Trophic patterns could be useful in terms of choosing priority targets for therapy: antiangiogenic drugs (for vascular type) or proliferation inhibitors (for proliferative type). The treatment and prognosis of adenocarcinoma with a mixed histological subtype is determined by the most aggressive component. Determining the trophic pattern could make it possible to personalize the selection of therapy, taking into account the peculiarities of the tumor’s metabolism and its interaction with the body. Solid-type tumors are more likely to have hypoxic zones, which makes them more sensitive to HIF inhibitors. The hypoxic pattern (solid subtype) underscores the link between impaired vascularization, central necrosis, and anaerobic metabolism. Local hypoxia may induce epithelial–mesenchymal transition, altering cell polarity and promoting therapy resistance. Additionally, the hypoxic microenvironment facilitates immune evasion by suppressing T-cell activity and upregulating PD-L1 expression [25]. These findings suggest potential for combination therapies targeting both antiangiogenic pathways (vascular endothelial growth factor—VEGF, HIF-1α) and immune checkpoints.

The initial segmentation–classification pipeline exhibited limitations in analyzing heterogeneous tumors. Future work will employ pixel-by-pixel segmentation using generative adversarial networks to improve the identification of regions with distinct vascularization and mitotic patterns. One key unresolved issue is the prognostic role of atypical mitoses in assessing lung cancer aggressiveness. While our study accounted for total mitotic count, morphological anomalies (e.g., multipolar or asymmetric divisions) may serve as additional prognostic markers. However, detecting these anomalies using standard histological methods is challenging, necessitating the development of machine learning algorithms for automated classification. Further training of the model with annotated atypical mitoses could clarify their relationship with survival, molecular genetics, and potential targets for antiproliferative therapy, based on blocking peptidyl arginine deiminases genes [26].

We acknowledge the limitations of our initial segmentation–classification pipeline, particularly in highly heterogeneous tumors. Future work will employ advanced deep learning architectures, such as generative adversarial networks, for pixel-by-pixel segmentation to better delineate regions with distinct trophic features. Furthermore, while our model quantified total mitotic count, the prognostic role of atypical mitotic figures remains an open question. The most important limitation of this study is that it is not prospective, meaning that our findings only indicate interesting possibilities for personalized treatment strategies. To prove the effectiveness of computer vision-driven approaches, a pilot clinical study is required. In our future research, we intend to use a combined analysis of mitoses and vasculature to identify groups of patients with high probabilities of response to antiangiogenic therapy. The concept of trophic patterns, which integrates assessments of tumor proliferation and vascularization, offers a data-driven perspective on the biology of lung adenocarcinoma.

## 5. Conclusions

In this study, we have established a novel, data-driven taxonomy of trophic patterns in lung adenocarcinoma. These patterns provide a deeper, more functional understanding of subtype-specific biology beyond conventional histology.

The critical next step is to validate these patterns in prospective cohorts with linked clinical and outcome data. Therefore, our future research will focus on correlating these defined trophic patterns with responses to antiangiogenic therapy, immunotherapy, and overall survival. This will determine the ultimate prognostic and predictive power of our approach, moving it from a biological insight towards a potential tool for personalized oncology.

## Figures and Tables

**Table 1:** Vessel dataset composition.

Dataset	Data Format	WSI Count
NLST	SVS	57
DHMC	TIFF	77
TCGA-LUAD	SVS	9
OWN_DB	SVS, TIFF	56

**Table 2:** False positive reduction effect on metrics.

Quality Metrics of the DHMC Dataset Before and After Model Tuning						
**Before tuning**						
**Negative class**	**TP**	**FP**	**TN**	**FN**	**Sensitivity**	**Specificity**
Ink (4)	193	5	294	15	0.92	0.98
Muscle/fibroblast Nucleus (5)	193	240	19	15	0.92	0.07
Chromatin (6)	193	80	6	15	0.92	0.06
4 + 5 + 6	193	325	319	15	0.92	0.49
**After tuning**						
**Negative class**	**TP**	**FP**	**TN**	**FN**	**Sensitivity**	**Specificity**
Ink (4)	182	2	297	26	0.97	0.99
Muscle/fibroblast Nucleus (5)	182	1	258	26	0.97	0.99
Chromatin (6)	182	21	65	26	0.97	0.76
4 + 5 + 6	182	24	620	26	0.97	0.96

## Data Availability

The source code is available from the corresponding author upon reasonable request for academic and non-commercial purposes. A detailed description of the model architecture, training procedures, hyperparameters, and data splits is provided in the Methods Section to facilitate reproducibility.

## Abbreviations

The following abbreviations are used in this manuscript:

AI	Artificial intelligence
DHMC	Dartmouth-Hitchcock Medical Center
EGFR	Epidermal growth factor receptor
FP	False positive
HIF	Hypoxia-inducible factor
NLST	National Lung Screening Trial
OWN_D	Own dataset
PD-L1	Programmed death-ligand 1
TCGA	The Cancer Genome Atlas Lung Adenocarcinoma
TP	True positive
VEGF	Vascular endothelial growth factor—VEGF
WSI	Whole-slide image

## Appendix A

**Table A1:** Quantitative morphological features of lung adenocarcinoma vessels and mitoses.

Name of the Parameter	Symbol	Dimension	Role of the Parameter Value
Area of tumor tissue	TA	µm^2^	Tumor tissue area on WSI. Helps to evaluate the stage
Area of tumor side tissue	TS	µm^2^	The area of adjacent non-tumor tissue. The size of the tumor microenvironment on WSI is important to assess the effect of a tumor on side tissues
Number of vessels	N_vessels	units	Number of tumor and tumor side vessels. Basic angiogenesis score
Tumor area small vessels	tumor_area_vessels_small_cnt	units	A total number of tumor small vessels in WSI
Tumor area big vessels	tumor_area_vessels_big_cnt	units	A total number of tumor big vessels on WSI
Tissue small vessels	tissue_area_vessels_small_cnt	units	A total number of tissue small vessels on WSI
Tissue big vessels	tissue_area_vessels_big_cnt	units	A total number of tissue big vessels on WSI
Area of vessels (in tumor)	A_vessels	µm^2^	Total area of tumor vessels provides information about general vascularization
Number of invasion vessels	N_invasive	units	Number of vessels with LVI features. A prognostic marker of future metastases
Number of mitotic figures (in tumor)	N_mitosis	units	Shows the activity of cell proliferation. The higher the number, the higher the rate of tumor growth
Feret diameter of vessels	FD	µm	Describes the geometric shape of the vessel

**Table A2:** Vascularization-proliferation metrics, based on parameters from Table A1.

**Name of the Metric**	**Abbreviation**	**Formula/Dimension**	**Role of the Metric Value**
Vessels number density (in tumor)	VND	$VND\;=\;\frac{N\_vessels\;}{TA}$, vessels/µm^2^	The number of vessels per unit area of the tumor. Assessment of perfusion level
Vessels relative area density (in tumor)	VAD_TUMOR	$VAD\;=\;\frac{A\_vessels\;}{TA}$, % (×100)	The proportion of the tumor area with blood vessels. Assessment of relative vascularization
Vessels relative area density (in tissue)	VAD_TISSUE	$VAD\;=\;\frac{A\_vessels\;}{TS}$, % (×100)	The proportion of the tumor side with blood vessels. Assessment of relative vascularization
Peripheral/central ratio for small vessels	tumor_area_vessels_small_supply_ratio	tissue_area_vessels_small_cnt/tumor_area_vessels_small_cnt	The predominance of vessels on the tumor side may indicate active angiogenesis at the edges, which is typical for aggressive tumors
Peripheral/central ratio for big vessels	tumor_area_vessels_big_supply_ratio	tissue_area_vessels_big_cnt/tumor_area_vessels_big_cnt	The predominance of vessels on the tumor side may indicate active angiogenesis at the edges, which is typical for aggressive tumors
Vessels invasion index	VII	$VII\;=\;\frac{N\_invasive\;}{N\_vessels\;}$, % (x100)	The proportion of vessels with LVI features among all vessels. Indicates the risk of tumor metastasis
Mitoses number (in tumor)	MD N_mitoses/A_Tumor)	$MD\;=\;\frac{N\_mitosis\;}{TA}$, mitosis/µm^2^	The number of mitoses per unit area of the tumor. Indicates tumor growth points
Shannon entropy (in tumor)	SE	$VAD\;=\;\sum_{i=1}^{K}p_{i}{log}_{2}p_{i}$ where -K—number of spatial segments (for example, grid cells or clusters), -$p_{i}$—the relative frequency of mitosis in the I-th segment, dits	It shows how evenly or focused the mitotic foci are. Identification of “hot spots” (clusters) of aggressive growth
Fractal size of mitosis (in tumor)	FS	Method box-counting	Spatial estimation of the distribution of mitotic figures. High fractality indicates active branched tumor growth and allows us to evaluate this proliferation metric on a small amount of material.
Mitosis clusters	mitosis_dbscan_clusters	DBSCAN	The presence of mitotic clusters may indicate local foci of high proliferative activity
«Center-to-edge» symmetry coefficient	Sym_C	$Sym\_C=\frac{{VCD\;}_{center}}{{VCD\;}_{edge}}$	The ratio of vascular densities in the tumor center and on the tumor side. An indicator of asymmetric growth

**Table A3:** Difference between column means for each trophic metric and parameter.

**Metric/Parameter**	**Predicted Mean of** **Native**	**Predicted** **Mean of Clean**	**Difference Between** **Predicted** **Means**	**Standard Error** **of** **Difference**	**95% CI of** **Difference**
Mitoses entropy	2.76	2.96	−0.20	0.17	−0.53 to 0.13
Vessels relative area density in tumor	0.03	0.03	0.00	0.00	0.00
Number of small vessels in the tumor center	16.54	16.10	0.44	2.47	−4.41 to 5.28
Number of small vessels in the tumor periphery	68.20	64.18	4.02	9.15	−13.96 to 21.99
Number of large vessels in the tumor center	6.54	6.40	0.14	0.89	−1.62 to 1.91
Number of large vessels in the tumor periphery	23.41	22.98	0.44	3.37	−6.19 to 7.06
